# NMD monitors translational fidelity 24/7

**DOI:** 10.1007/s00294-017-0709-4

**Published:** 2017-05-23

**Authors:** Alper Celik, Feng He, Allan Jacobson

**Affiliations:** Department of Microbiology and Physiological Systems, University of Massachusetts Medical School, 368 Plantation Street, Worcester, MA 01655 USA

**Keywords:** NMD substrates, Translational fidelity, Frameshifting, Probabilistic mRNA decay

## Abstract

Nonsense-mediated mRNA decay (NMD) is generally thought to be a eukaryotic mRNA surveillance pathway tasked with the elimination of transcripts harboring an in-frame premature termination codon (PTC). As presently conceived, NMD acting in this manner minimizes the likelihood that potentially toxic polypeptide fragments would accumulate in the cytoplasm. This notion is to be contrasted to the results of systematic RNA-Seq and microarray analyses of NMD substrates in multiple model systems, two different experimental approaches which have shown that many mRNAs identified as NMD substrates fail to contain a PTC. Our recent results provide insight into, as well as a possible solution for, this conundrum. By high-resolution profiling of mRNAs that accumulate in yeast when the principal NMD regulatory genes (*UPF1, UPF2,* and *UPF3*) are deleted, we identified approximately 900 NMD substrates, the majority of which are normal-looking mRNAs that lack PTCs. Analyses of ribosomal profiling data revealed that the latter mRNAs tended to manifest elevated rates of out-of-frame translation, a phenomenon that would lead to premature translation termination in alternative reading frames. These results, and related observations of heterogeneity in mRNA isoforms, suggest that NMD should be reconsidered as a probabilistic mRNA quality control pathway that is continually active throughout an mRNA’s life cycle.

NMD is known as an mRNA surveillance mechanism, but until recently a coherent understanding of the targets of its surveillance activity has been difficult to pin down. Initially characterized in yeast and worms (Leeds et al. 1991; Peltz et al. 1993; Pulak and Anderson 1993), NMD was first thought to selectively degrade mRNAs transcribed from nonsense or frameshift alleles to diminish the possible dominant-negative effects of truncated polypeptides. This simple functional model was rapidly broadened to a more general role in mRNA quality control with the recognition that the pathway’s substrates included unspliced pre-mRNAs that had entered the cytoplasm (He et al. 1993; Pulak and Anderson 1993; Sayani et al. 2008), products of alternative splicing (Jaillon et al. 2008; Lareau et al. 2007; Lykke-Andersen et al. 2014; Ni et al. 2007), transcripts of pseudogenes or unproductive gene rearrangements (He et al. 2003; Li and Wilkinson 1998; McGlincy and Smith 2008), mRNAs subject to programmed frameshifting or leaky scanning (He et al. 2003; Welch and Jacobson 1999), and mRNAs with upstream open reading frames (uORFs) (Arribere and Gilbert 2013; Gaba et al. 2005; He et al. 2003). In all such cases, it was easy to rationalize these additional substrates because their translation would ultimately lead to a ribosome’s encounter with a PTC. However, the advent of genome-wide microarray and RNA-Seq analyses allowed for the comprehensive assembly of catalogs of NMD substrates in multiple organisms and these studies showed that NMD targets large numbers of apparently normal wild-type mRNAs (He et al. 2003; Lelivelt and Culbertson 1999; Rehwinkel et al. 2005). For example, our RNA-Seq analyses of mRNAs differentially expressed in yeast cells lacking the principal NMD regulators Upf1, Upf2, or Upf3 identified approximately 900 commonly upregulated mRNAs, of which the vast majority were normal-looking transcripts with complete open reading frames (Celik et al. 2017).

Clearly, the existence of so many apparently PTC-free substrates has been perplexing and led to speculation that NMD had somehow been co-opted to regulate the levels of expression of seemingly normal genes. We believe that we have now resolved this conundrum. By analyzing ribosomal profiling data for yeast mRNAs that are NMD or non-NMD substrates, we found that the normal-looking yeast NMD substrates have significantly lower ribosome densities throughout their open reading frames than the non-substrates, i.e., although these mRNAs appear normal they are translated relatively poorly (Celik et al. 2017). Further, contrary to earlier hypotheses that Upf1, the central regulator of NMD, might silence the translation of NMD-targeted mRNAs (Isken et al. 2008; Muhlrad and Parker 1999), these deficiencies in translation are independent of the presence of Upf1. Most importantly, when compared to non-NMD substrates, the normal-looking NMD substrates were found to have a higher rate of out-of-frame translation, lower average codon optimality, and a propensity to have longer stretches of non-optimal codons (Celik et al. 2017).

The implications of these observations are far reaching for our understanding of the cellular role of NMD, and its mechanism of activation. First, NMD targeting of the large number of normal-looking mRNAs may be caused by the decoding events occurring during a run of non-optimal codons. This could account for the diminished efficiency of translation for these transcripts, an increased probability for translational elongation errors, and, as a consequence, an enhanced rate of out-of-frame translation accompanied by a high likelihood encounter with an out-of-frame nonsense codon and ensuing premature termination (Fig. 1). Second, while it is possible that the reduced efficiency of translation and enhanced rate of out-of-frame translation detected for the normal-looking NMD substrates could have other causes, they appear less likely than those associated with sub-optimal translation. For example, the normal-looking NMD substrates may each have multiple transcript isoforms, possibly resulting from transcriptional initiation within protein coding regions (Malabat et al. 2015). Such isoforms might lack the normal 5′-UTRs and initiation codons and instead utilize downstream out-of-frame AUGs for translation initiation. Alternative splicing events might also yield a subset of NMD-targeted transcripts. However, the elevated rates of out-of-frame translation observed with normal-looking NMD substrates are largely independent of iTSS status (Celik et al. 2017) and only minor mRNA isoforms, i.e., those unlikely to have significant impact on transcriptome-wide studies, are thought to be generated by alternative splicing events in yeast (Kawashima et al. 2014). Third, with a propensity for frameshifting the most likely basis for premature termination and NMD substrate status of the normal-looking mRNAs, it is time to think of NMD as a probabilistic quality control mechanism, i.e., one that is capable of constant monitoring of gene expression errors that affect maintenance of the normal reading frame during mRNA translation. As summarized in Fig. 2, NMD substrates must encompass not only those mRNAs in which a ribosome’s encounter with a PTC is obvious and hard-wired (“traditional” NMD substrates), but also those mRNAs in which non-standard transcription initiation, downstream translation initiation, or unexpected frameshifting lead to premature translation termination, i.e., termination upstream of the site normally used for a given ORF (“probabilistic” NMD substrates). Finally, since the underlying principle of probabilistic decay implies that NMD can occur at any time during an mRNA’s translational life cycle it’s important to reconsider the popular “pioneer round” model for NMD (Maquat 2004). Although this model posits that NMD, at least in metazoans, only occurs during the initial round of mRNA translation, the results summarized here, as well as experiments in both yeast and mammalian cells showing that steady-state mRNAs can be targeted by NMD (Durand and Lykke-Andersen 2013; Gaba et al. 2005; Maderazo et al. 2003; Rufener and Muhlemann 2013) all indicate that the presence of a PTC in an elongating ribosome’s A site will almost always trigger NMD and that mRNAs undergoing translation cannot acquire immunity from NMD. This, of course, is reassuring when NMD is thought of as a cellular quality control mechanism that minimizes the accumulation of potentially toxic polypeptide fragments. Given the high degree of conservation of the NMD regulators it is likely that the principles governing NMD substrate status that appear to be operational in yeast hold for all eukaryotes.

**Fig. 1:**
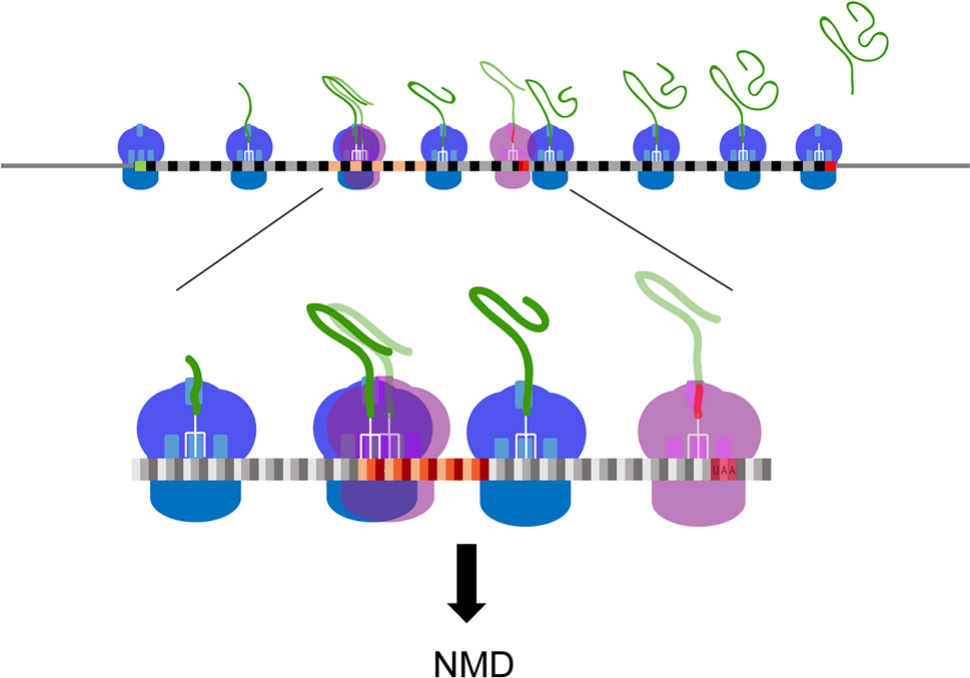
Ribosomal failure to maintain the correct mRNA reading frame is a common basis for NMD targeting. The figure depicts translation of a “normal-looking” mRNA and the ribosomal elongation events that lead to its acquisition of NMD substrate status. *Top: blue* ribosomes are translating the mRNA in the annotated open reading frame (*gray* and *black boxes*) whereas the *pink ribosomes* have entered a stretch of non-optimal codons (*pink*, *orange*, and *red boxes*) and shifted to the +1 reading frame. *Bottom*: higher resolution depiction of a premature termination event that occurs as a consequence of ribosomal elongation in the +1 reading frame

**Fig. 2:**
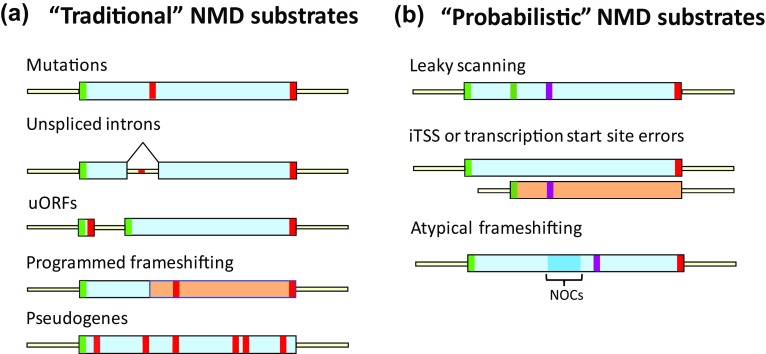
NMD targets distinct types of substrates. mRNAs become substrates for NMD when an elongating ribosome encounters a PTC that has been generated in one of several different ways. **a** “Traditional” NMD substrates. For these mRNAs, translation begins at initiation codons located at ORF (or uORF) 5′ ends, and elongation then proceeds 3′, leading to ribosomal A site positioning of an in-frame PTC. Substrates in this class include mRNAs derived from nonsense alleles, intron-containing pre-mRNAs that enter the cytoplasm, uORF-containing mRNAs, mRNAs in which programmed frameshifting allows a fraction of ribosomes to avoid premature termination, and mRNAs transcribed from pseudogenes. **b** “Probabilistic” NMD substrates. These mRNAs lack in-frame PTCs in their annotated ORFs, but contain features that promote either downstream out-of-frame translational initiation or frameshifting, thus leading to premature translation termination in a new reading frame. mRNAs in this category can have poor sequence context around the normal initiation codon, a transcription start site that is internal to the principal ORF, lower overall codon optimality, or a long stretch of non-optimal codons (NOCs). In each example, a subset of ribosomes translates the mRNA in a frame different from that of the annotated ORF. *Green* initiation codon; *red* stop codon; *yellow* UTR; *purple* stop codon encountered in the +1 or +2 reading frame; *blue* cluster of non-optimal codons. From Celik et al. (2017)
